# Supplementary material for: GhCYP710A1 Participates in Cotton Resistance to Verticillium Wilt by Regulating Stigmasterol Synthesis and Plasma Membrane Stability
Source: Int J Mol Sci. 2022 Jul 29;23(15):8437. doi: 10.3390/ijms23158437 (PMC9368853; doi:10.3390/ijms23158437)
Supplement: Supplementary file 1 [file ijms-23-08437-s001.zip › The legend of Figure S1 and Figure S2.pdf]

Figure S1 Identification of the *GhCYP710A1*-overexpresion transgenic cotton

(A) GUS staining results of *GhCYP710A1*-overexpression transgenic cotton. (B) PCR amplification results of *GhCYP710A1*-overexpression transgenic cotton, primers are gene upstream primer (*GhCYP710A1*-R) with NOS terminal primer; M: DNA Marker. P: positive control, template is sense *GhCYP710A1* plasmid; N: negative control, template is gDNA of wild-type cotton leave; W: blank control, water is template; OE-1~OE-8 represent different transgenic cottons. (C) Relative expression levels of *GhCYP710A1* in leaves of transgenic cotton. Error bars represent SD of three independent replicates. ** represent $P<0.01$, *** represent $P \leqslant 0.001$.

Figure S2 Identification of VIGS-induced *GhCYPA710A1* gene silencing in cotton

(A) Diagram of TRV: *GhCYP710A1* vector. LB: The left border of T-DNA; CaMV35S: promoter; RB: The right border of T-DNA; TRV-RNA2 5′: 5′ noncoding region; TRV-RNA2 3′: 3′ noncoding region; Nos-T: terminator; EcoRI and BcuI indicate multiple cloning sites for *GhCYP710A1* insertion. (B) Silencing *GhCYP710A1* cotton phenotype. TRV:00 represent negative control of pTRV1+pTRV2. (C) Relative transcription level of *GhCYP710A1* gene in TRV: *GhCYP710A1* plants. Error bars represent the standard deviation of three independent replicates.
