# Supplementary figures and images for: GhCYP710A1 Participates in Cotton Resistance to Verticillium Wilt by Regulating Stigmasterol Synthesis and Plasma Membrane Stability
Source: Int J Mol Sci. 2022 Jul 29;23(15):8437. doi: 10.3390/ijms23158437 (PMC9368853; doi:10.3390/ijms23158437)

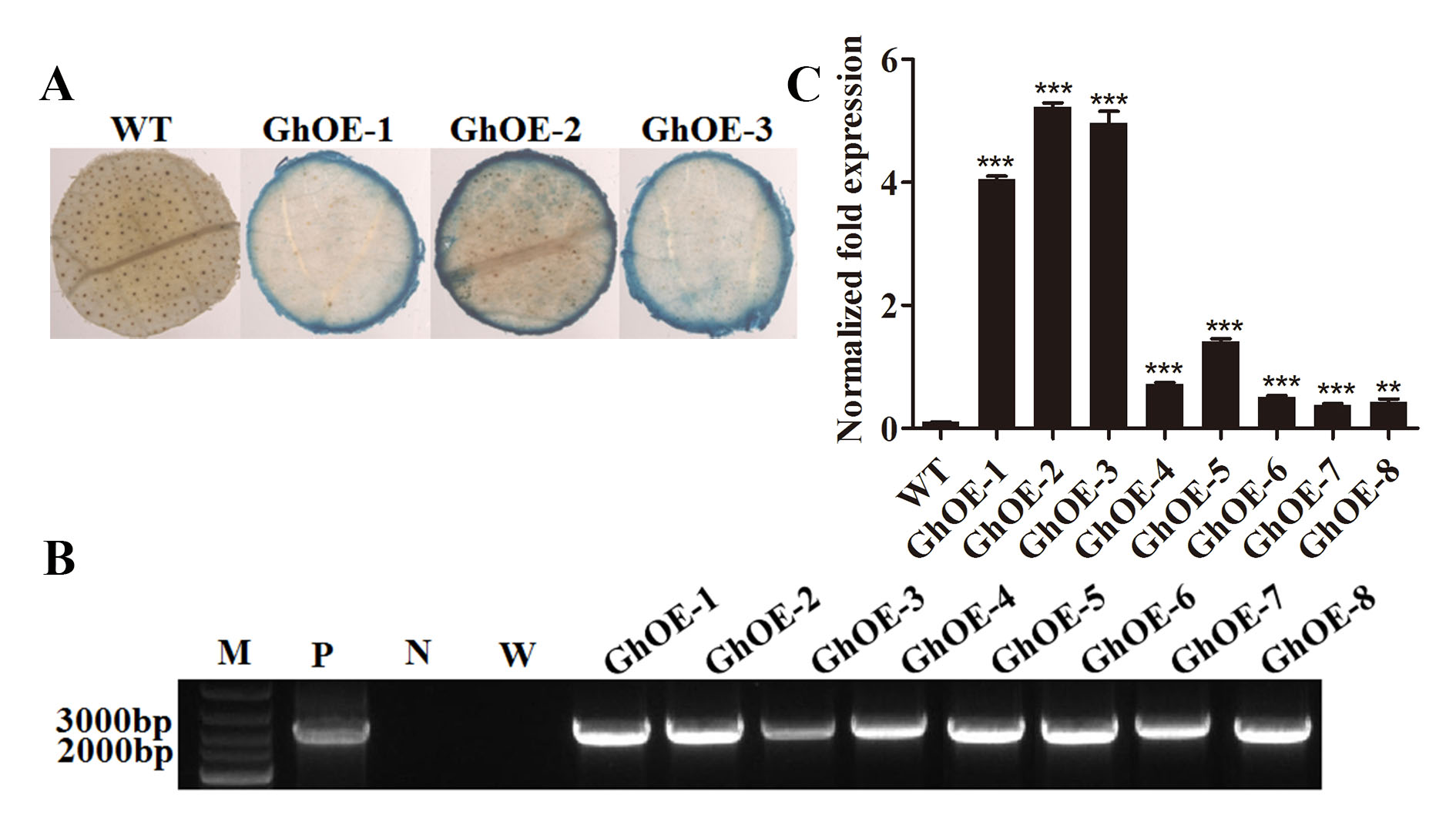

Supplement: Supplementary file 1 [file ijms-23-08437-s001.zip › Figure S1.jpg]

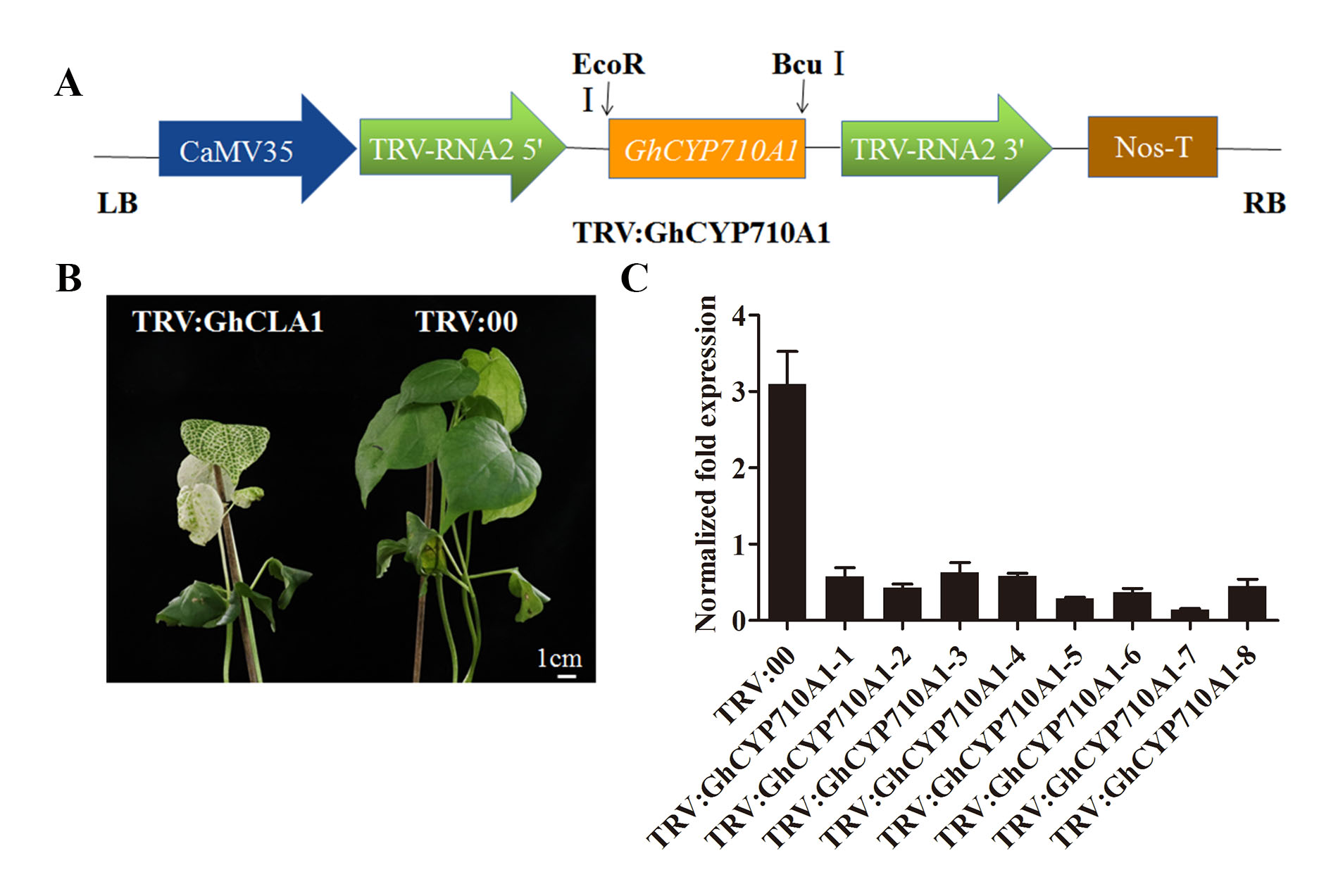

Supplement: Supplementary file 1 [file ijms-23-08437-s001.zip › Figure S2.jpg]
